# Supplementary material for: Bioinformatic Workflows for Generating Complete Plastid Genome Sequences—An Example from Cabomba (Cabombaceae) in the Context of the Phylogenomic Analysis of the Water-Lily Clade
Source: Life (Basel). 2018 Jun 21;8(3):25. doi: 10.3390/life8030025 (PMC6160935; doi:10.3390/life8030025)
Supplement: Supplementary file 1 [file life-08-00025-s001.zip › life-305093.R1_RevisedManuscript_SupplementaryFiles/SupplementaryFile10_Script10.pdf]

```
#!/bin/bash

# AUTHOR="Michael Gruenstaeudl, PhD"
# COPYRIGHT="Copyright (C) 2016-2018 $AUTHOR"
# CONTACT="m.gruenstaeudl@fu-berlin.de"
# VERSION="2018.04.30.1600"
# USAGE="bash Script10.sh $INF_ALGNM $LOG"

########################################################################
# SUPPLEMENTARY FILE 10                                                #
# Bash script to remove gap positions and to calculate alignment       #
# statistics from an input alignment.                                  #
########################################################################

# Check if sufficient commandline parameters
numArgmts=$#
if [ ! $numArgmts -eq 2 ]; then
    echo "ERROR | Incorrect number of commandline parameters" >&2
    exit 1
fi

# Check if input files exist
for v in "$@"; do
if [ ! -f "$v" ]; then
    echo "ERROR | File not found: $v" >&2
    exit 1
fi
done

# Check if dependencies exist
DEPS=(statal trimal)
for d in "${DEPS[@]}"; do
if ! [ -x "$(command -v $d)" ]; then
  echo "Error: $d is not installed" >&2
  exit 1
fi
done

# Assigning commandline arguments
INF_ALGNM=$1
LOG=$2

########################################################################

# Defining temporary files and outfiles
TMP_ALGNM=${INF_ALGNM%.nex*}.tmp
OUF_ALGNM=${INF_ALGNM%.nex*}_gapsRemoved.nex

####################################
## Replace question marks with Ns ##
####################################
cat $INF_ALGNM | tr '?' 'N' > $TMP_ALGNM

######################################
## Operations via trimal and statal ##
######################################

echo -e "\n# Alignment statistics BEFORE gap removal" >> $LOG
statal -in $TMP_ALGNM -sident >> $LOG
statal -in $TMP_ALGNM -sgt >> $LOG

trimal -in $TMP_ALGNM -out $OUF_ALGNM -fasta -gt 0.8 -st 0.001 -cons 60

echo -e "\n# Alignment statistics AFTER gap removal" >> $LOG
statal -in $OUF_ALGNM -sident >> $LOG
statal -in $OUF_ALGNM -sgt >> $LOG

# File hygiene
rm $TMP_ALGNM
```
