# Supplementary material for: Bioinformatic Workflows for Generating Complete Plastid Genome Sequences—An Example from Cabomba (Cabombaceae) in the Context of the Phylogenomic Analysis of the Water-Lily Clade
Source: Life (Basel). 2018 Jun 21;8(3):25. doi: 10.3390/life8030025 (PMC6160935; doi:10.3390/life8030025)
Supplement: Supplementary file 1 [file life-08-00025-s001.zip › life-305093.R1_RevisedManuscript_SupplementaryFiles/SupplementaryFile11_Script11.pdf]

```
#!/usr/bin/Rscript

# AUTHOR="Michael Gruenstaeudl, PhD"
# COPYRIGHT="Copyright (C) 2016-2018 $AUTHOR"
# CONTACT="m.gruenstaeudl@fu-berlin.de"
# VERSION="2018.06.09.1500"
# USAGE="R Script11.R $FINAL_ALGNM"

########################################################################
# SUPPLEMENTARY FILE 11                                                #
# R script to infer the best phylogenetic tree under the maximum       #
# likelihood criterion given a DNA alignment, and to infer node        #
# support for the best ML tree via bootstrapping.                      #
########################################################################

#####################
# LOADING LIBRARIES #
#####################
library(ape)
library(phangorn)
library(tools) # For function 'file_path_sans_ext'
library(svglite) # For improved svg drivers

####################
# FUNCTIONS #
####################

find_max_lik_tree = function(alignm, nuclsubmodel) {
    ## ARGS:
    ##  alignm (list): a DNA sequence alignment
    ##  nuclsubmodel (string) = the best-fitting nucleotide substitution model for t
he alignment
    ## RETURN:
    ##  BestMLtree_withBSvalues (a tree object)
    ## HELP:
    ##  See document "Estimating phylogenetic trees with phangorn" by Klaus P. Schli
ep

    # Split nucleotide substitution model into palpatable pieces
    model_hndl = unlist(strsplit(nuclsubmodel, '\\+'))
    model = model_hndl[1]
    optInv = FALSE
    optGamma = TRUE
    if (length(model_hndl)==2 & model_hndl[2]=='G') {optGamma=TRUE}
    if (length(model_hndl)==2 & model_hndl[2]=='I') {optInv=TRUE}
    if (length(model_hndl)==3) {optGamma=TRUE; optInv=TRUE}

    # Compute distances between DNA sequences, using a simple empirical model
    dm = dist.ml(alignm, model='F81')
    # Calculate NJ tree as starting tree
    treeNJ = NJ(dm)
    # Compute the likelihood of the start tree
    fitStart = pml(treeNJ, data=alignm)
    # Optimize branch lengths under best-fiting nucleotide substitution model
    #fitBest = optim.pml(fitStart, model=model, optInv=optInv, optGamma=optGamma, re
arrangement='stochastic')
    fitBest = optim.pml(fitStart, model=model, optInv=optInv, optGamma=optGamma, rea
rrangement='NNI')
    # Apply bootstrap to evaluate how well the nodes of the trees are supported
    BSvalues = bootstrap.pml(fitBest, bs=1000, optNni=TRUE, multicore=TRUE)
    # Plot most likely tree with bootstrap values
    #BestMLtree_withBSvalues = plotBS(fitBest$tree, BSvalues, p = 50, type="p")
    BestMLtree_withBSvalues = plotBS(fitBest$tree, BSvalues, p = 50, type="u")

    # Prepare output
    output = list()
    output[["tree"]] = BestMLtree_withBSvalues
    output[["lnL"]] = fitBest$logLik
    output[["g"]] = fitBest$g
    output[["inv"]] = fitBest$inv
    # Return output
    return(output)
}
```

```
########
# MAIN #
########

# SPECIFYING INFILES
cmdArgs = commandArgs(trailingOnly = TRUE)
inFile = cmdArgs

# SPECIFYING OUTFILES
outFile_stem = file_path_sans_ext(inFile)
outFile_tre = paste(outFile_stem, '.tre', sep='')
outFile_svg = paste(outFile_stem, '.svg', sep='')

# SPECIFY NUCLEOTIDE SUBSTITUTION MODEL
nuclsubmodel = "GTR+I+G"

# LOAD ALIGNMENT
# For ML tree inference via phangorn (see function 'findmaxliktree.R'), alignments m
ust be read via read.phyDat
alignm = read.phyDat(inFile, format='fasta', type='DNA')

# INFER BEST ML TREE
output = tryCatch( # NOTE: A certain stochasiticity is underlying the ML tree infere
nce, which is why it sometimes fails and requires a tryCatch.
    expr = find_max_lik_tree(alignm, nuclsubmodel),
    error = function(e) {cat('FAIL\n'); return(NULL)}
    )

# SAVE TREES TO FILE
write.tree(output$tree, file=outFile_tre)

# PREPARE TREE STATS
lnL_value = paste("logLike value:", output$lnL)
gamma_value = paste("Gamma value:", output$g)
invSites_value = paste("InvSites value:", output$i)

# PLOT ML TREE WITH BS-VALUES AND SCALEBAR
svglite(outFile_svg, standalone=TRUE)
plotBS(output$tree)
# Add scalebar
add.scale.bar()
# Add tree stats
text(x=0, y=0, pos=1, labels=c(lnL_value, gamma_value, invSites_value))
dev.off()


# EOF
```
