# Supplementary material for: Bioinformatic Workflows for Generating Complete Plastid Genome Sequences—An Example from Cabomba (Cabombaceae) in the Context of the Phylogenomic Analysis of the Water-Lily Clade
Source: Life (Basel). 2018 Jun 21;8(3):25. doi: 10.3390/life8030025 (PMC6160935; doi:10.3390/life8030025)
Supplement: Supplementary file 1 [file life-08-00025-s001.zip › life-305093.R1_RevisedManuscript_SupplementaryFiles/SupplementaryFile12_Tutorial.pdf]

Scripts for Standardized Plastid Phylogenomics

Version: 2018.04.30, Author: Michael Gruenstaeudl

Software scripts to automate and standardize the basic bioinformatic processes in plastid phylogenomics

GETTING STARTED

File prerequisites

- Raw reads from Illumina platform (R1 and R2, in separate files)
- Control and sample number of your Illumina run (i.e., the last two fields of a FASTQ header)
  - Note: For more info the format of a FASTQ header, see here
- One or more plastid genomes that can be used as reference genomes (in a single FASTA-file)

Software prerequisites

The following software must available on your system:

- Git (for installation)
- bioawk (for SCRIPT 01)
- FASTX Toolkit (for SCRIPT 02)
- Python 2.7 (for SCRIPTS 03,07,09)
- IOGA (for SCRIPT 03)
- blastn (for SCRIPT 04)
- bowtie2 (for SCRIPTS 05,06)
- samtools (for SCRIPTS 05,06)
- bedtools (for SCRIPTS 05,06)
- Perl 5 (for SCRIPT 07)
- trimAl (for SCRIPT 10)
- R 3.4 (for SCRIPT 11)

INSTALLATION

Cloning this GitHub repository

```
cd /path_to_git/
git clone
https://github.com/michaelgruenstaeudl/StandardizedPlastidPhylogenomics.git
```

USAGE

1. Specifying file names and locations

Specifying the working directory and the local Git repository

```
MYWD=/path_to_infiles/
```

```
MYGIT=/path_to_git/StandardizedPlastidPhylogenomics/
```

Specifying sample name, reference name and FASTQ header

```
MYSAMPLE="Sample_name_here"  # Example: MYSAMPLE="NY684"
MYREF="Reference_name_here"  # Example: MYREF="MG720559"
FASTQ_HEADR="controlNum_sampleNum_here"  # Example: FASTQ_HEADR="N:0:6"
```

Specifying and initializing LOG-file

Adding the project name as the first line of the log-file.

```
LOG=$MYWD/${MYSAMPLE}.log
echo "$MYSAMPLE" > $LOG
```

2. Quality filtering of reads

SCRIPT 01 - Generating ordered intersection of reads

Bash script to generate an ordered intersection of paired-end Illumina reads and to quantify the loss of reads during this process.

```
## DECLARING VARIABLES
INF1=$MYWD/${MYSAMPLE}_R1.fastq
INF2=$MYWD/${MYSAMPLE}_R2.fastq

## RUNNING SCRIPT
bash $MYGIT/Script01.sh $INF1 $INF2 $LOG $FASTQ_HEADR
```

SCRIPT 02 - Conducting quality filtering of reads

Bash script to conduct quality filtering of Illumina reads and to quantify the loss of reads during quality filtering.

```
## DECLARING VARIABLES
INF1=$MYWD/${MYSAMPLE}_R1.intersect.fastq
INF2=$MYWD/${MYSAMPLE}_R2.intersect.fastq

## RUNNING SCRIPT
bash $MYGIT/Script02.sh $INF1 $INF2 $LOG
```

3. Plastid genome assembly

SCRIPT 03 - Conducting the genome assembly process

Bash script to automate the genome assembly process and to quantify assembly success.

```
## DECLARING VARIABLES
INF1=$MYWD/${MYSAMPLE}_R1.Q30filt.fastq
```

```
INF2=$MYWD/${MYSAMPLE}_R2.Q30filt.fastq
IOGA=/path_to_IOGA/IOGA.py
REF_DB=/path_to_reference_genomes/combined_references.fas

## RUNNING SCRIPT
bash $MYGIT/Script03.sh $INF1 $INF2 $LOG $IOGA $REF_DB $MYSAMPLE
```

Manual step - Stitching of contigs to complete genome

Recommendation: Use Geneious for this step

4. Evaluation of assembly

SCRIPT 04 - Confirming the IR boundaries

Bash script to automate the confirmation of IR boundaries via self-blasting.

```
## DECLARING VARIABLES
FINAL_ASMBLY=$MYWD/${MYREF}.fasta

## RUNNING SCRIPT
bash $MYGIT/Script04.sh $FINAL_ASMBLY $LOG
```

SCRIPT 05 - Extracting reads that map to final assembly, Generating assembly statistics - part 1

Bash script to automatically map the quality-filtered reads against the final assembly and to extract the mapped paired reads from the quality-filtered read files. This script also compiles a series of mapping statistics to describe the mapping process.

```
## DECLARING VARIABLES
INF1=$MYWD/${MYSAMPLE}_R1.Q30filt.fastq
INF2=$MYWD/${MYSAMPLE}_R2.Q30filt.fastq
FINAL_ASMBLY=$MYWD/${MYREF}.fasta

## RUNNING SCRIPT
bash $MYGIT/Script05.sh $INF1 $INF2 $FINAL_ASMBLY $LOG $MYSAMPLE
```

SCRIPT 06 - Generating assembly statistics - part 2

Bash script to automatically generate assembly quality statistics (i.e., the mean read length of mapped reads and the number of nucleotides with coverage depth equal or greater than 20, 50 and 100).

```
## DECLARING VARIABLES
INF_MAPPED_R1=$MYWD/${MYSAMPLE}.MappedAgainst.${MYREF}_R1.fastq
INF_MAPPED_R2=$MYWD/${MYSAMPLE}.MappedAgainst.${MYREF}_R2.fastq
INF_SAM=$MYWD/${MYSAMPLE}.MappedAgainst.${MYREF}.sam

## RUNNING SCRIPT
bash $MYGIT/Script06.sh $INF_MAPPED_R1 $INF_MAPPED_R2 $INF_SAM $LOG
```

5. Plastid genome annotation

Manual step - Plastid genome annotation via DOGMA and cpGAVAS

- Annotation server DOGMA
  - Note: Save the annotations of DOGMA as "Plain Text Summary"
- Annotation server cpGAVAS

SCRIPT 07 - Convert DOGMA plain text summary to GFF

Bash script to convert the plain text summary of the annotations generated by DOGMA to a GFF file.

```
## DECLARING VARIABLES
INF=$MYWD/${MYSAMPLE}_DOGMA_Annotations_PlainTextSummary.txt
FINAL_ASMBLY=$MYWD/${MYREF}.fasta

## RUNNING SCRIPT
bash $MYGIT/Script07.sh $INF $FINAL_ASMBLY
```

SCRIPT 08 - Combine annotations into single set and generate union set of the annotations

Bash script to combine the annotations produced by the annotation servers DOGMA and cpGAVAS and to generate a union set of the combined annotations.

```
## DECLARING VARIABLES
INF_CPGAVAS=$MYWD/${MYSAMPLE}_cpGAVAS_Annotations.gff
INF_DOGMA=$MYWD/${MYSAMPLE}_DOGMA_Annotations_PlainTextSummary.gff
FINAL_ASMBLY=$MYWD/${MYREF}.fasta
IRB="start_of_IRb,end_of_IRb"  # Example: IRB="89435,114903"
IRA="start_of_IRa,end_of_IRa"  # Example: IRA="134019,159487"

## RUNNING SCRIPT
bash $MYGIT/Script08.sh $INF_CPGAVAS $INF_DOGMA $FINAL_ASMBLY $IRB $IRA
```

6. Alignment of coding regions

SCRIPT 09 - Extract and align coding regions

Python script to extract and align coding regions across a set of input genomes in GenBank format.

```
## DOWNLOADING SCRIPT 'align_back_trans' OF Galaxy ToolShed
wget
https://raw.githubusercontent.com/peterjc/pico_galaxy/master/tools/align_back_tran
s/align_back_trans.py

## INPUT DATA
# Save all plastid genomes for alignment into folder $MYWD

## RUNNING SCRIPT
python2 $MYGIT/Script09.py -i $MYWD -o $MYSAMPLE
```

SCRIPT 10 - Remove gap positions

Bash script to remove gap positions and to calculate alignment statistics from an input alignment.

```
## DECLARING VARIABLES
INF_ALGNM=$MYWD/${MYSAMPLE}_nucl_82combined.aligned.nex

## RUNNING SCRIPT
bash $MYGIT/Script10.sh $INF_ALGNM $LOG
```

7. Phylogenetic analysis

SCRIPT 11 - Phylogenetic tree inference under ML, including bootstrapping

R script to infer the best phylogenetic tree under the maximum likelihood criterion given a DNA alignment, and to infer node support for the best ML tree via bootstrapping.

```
## DECLARING VARIABLES
FINAL_ALGNM=$MYWD/${MYSAMPLE}_nucl_82combined.aligned_gapsRemoved.fas

## RUNNING SCRIPT
Rscript $MYGIT/Script11.R $FINAL_ALGNM
```

TEST DATA

Test data is available at: https://zenodo.org/record/1213269

CITATION

Gruenstaeudl M., Gerschler N., Borsch T. (2018) Sharing bioinformatic workflows for plastid genomics - An example from the sequencing of complete plastid genomes of *Cabomba* (Cabombaceae). In Review.

CHANGELOG

- 2018.04.30 - Update
    - Addition of SCRIPTS 09, 10, 11
- 2018.04.05 - Update
    - Addition of SCRIPT 07, update of SCRIPT 08
- 2018.04.04 - Update
    - Update of README
- 2018.04.03 - Update
    - Update of scripts
- 2018.02.28 - Original Upload
    - First upload of scripts
