# Supplementary material for: Bioinformatic Workflows for Generating Complete Plastid Genome Sequences—An Example from Cabomba (Cabombaceae) in the Context of the Phylogenomic Analysis of the Water-Lily Clade
Source: Life (Basel). 2018 Jun 21;8(3):25. doi: 10.3390/life8030025 (PMC6160935; doi:10.3390/life8030025)
Supplement: Supplementary file 1 [file life-08-00025-s001.zip › life-305093.R1_RevisedManuscript_SupplementaryFiles/SupplementaryFile1_Script01.pdf]

```
#!/bin/bash

# AUTHOR="Michael Gruenstaeudl, PhD"
# COPYRIGHT="Copyright (C) 2016-2018 $AUTHOR"
# CONTACT="m.gruenstaeudl@fu-berlin.de"
# VERSION="2018.04.03.1800"
# USAGE="Script1.sh $INF1 $INF2 $LOG $FASTQ_HEADR"

#############################################################################
# SUPPLEMENTARY FILE 1                                                      #
# Bash script to generate an ordered intersection of paired-end            #
# Illumina reads and to quantify the loss of reads through this            #
# process.                                                                  #
#############################################################################

# Check if sufficient commandline parameters
numArgmts=$#
if [ ! $numArgmts -eq 4 ]; then
    echo "ERROR | Incorrect number of commandline parameters" >&2
    exit 1
fi

# Check if input files exist
# ${@:1:3} means all input arguments except third one
for v in "${@:1:3}"; do
if [ ! -f "$v" ]; then
    echo "ERROR | File not found: $v" >&2
    exit 1
fi
done

# Check if dependencies exist
DEPS=(bioawk)
for d in "${DEPS[@]}"; do
if ! [ -x "$(command -v $d)" ]; then
  echo "Error: $d is not installed" >&2
  exit 1
fi
done

# Assigning commandline arguments
INF1=$1
INF2=$2
LOG=$3
FASTQ_HEADR=$4

# Defining temporary files and outfiles
INF1_HEAD=${INF1%.fastq*}.headers.tmp
INF2_HEAD=${INF2%.fastq*}.headers.tmp
INF1_SORT=${INF1%.fastq*}.sorted.tmp
INF2_SORT=${INF2%.fastq*}.sorted.tmp
OUF1=${INF1%.fastq*}.intersect.fastq
OUF2=${INF2%.fastq*}.intersect.fastq

# Counting reads prior to generating ordered intersection
VAR1=$(cat $INF1 | grep "^@M" | wc -l)
VAR2=$(cat $INF2 | grep "^@M" | wc -l)

# Logging results
echo -e "\n# NUMBER OF RAW READS" >> $LOG
echo -ne "$INF1: " >> $LOG
LC_ALL=C printf "%'d\n" $VAR1 >> $LOG
echo -ne "$INF2: " >> $LOG
LC_ALL=C printf "%'d\n" $VAR2 >> $LOG

# Sorting each read by read header
bioawk -c fastx '{print}' $INF1 | sort | awk -F'\t' '{print "@"$1;print $2;print "+"
$1;print $3}' > $INF1_SORT
bioawk -c fastx '{print}' $INF2 | sort | awk -F'\t' '{print "@"$1;print $2;print "+"
$1;print $3}' > $INF2_SORT

# Extracting sorted headers
bioawk -c fastx '{print}' $INF1 | sort | awk -F'\t' '{print "@"$1}' > $INF1_HEAD
```

```
bioawk -c fastx '{print}' $INF2 | sort | awk -F'\t' '{print "@"$1}' > $INF2_HEAD

# Removing pair info from headers
sed -i "s/\_1:$HEADR//g" $INF1_HEAD
sed -i "s/\_2:$HEADR//g" $INF2_HEAD

# Listing intersection elements
comm -12 $INF1_HEAD $INF2_HEAD > intersect.headers

# Saving only reads in intersection
grep --no-group-separator -A3 -Ff $INF1_HEAD $INF1_SORT > $OUF1
grep --no-group-separator -A3 -Ff $INF2_HEAD $INF2_SORT > $OUF2

# Counting reads after generating ordered intersection
VAR1=$(cat $OUF1 | grep "^@M" | wc -l)
VAR2=$(cat $OUF2 | grep "^@M" | wc -l)
if (($VAR1 == $VAR2)); then echo "SUCCESS"; else echo "FAIL"; fi

# Logging results
echo -e "\n# NUMBER OF READS IN ORDERED INTERSECTION" >> $LOG
echo -ne "$INF1: " >> $LOG
LC_ALL=C printf "%'d\n" $VAR1 >> $LOG
echo -ne "$INF2: " >> $LOG
LC_ALL=C printf "%'d\n" $VAR2 >> $LOG

# File hygiene
rm $INF1_HEAD
rm $INF2_HEAD
rm $INF1_SORT
rm $INF2_SORT
rm intersect.headers

#EOF
```
