# Supplementary material for: Bioinformatic Workflows for Generating Complete Plastid Genome Sequences—An Example from Cabomba (Cabombaceae) in the Context of the Phylogenomic Analysis of the Water-Lily Clade
Source: Life (Basel). 2018 Jun 21;8(3):25. doi: 10.3390/life8030025 (PMC6160935; doi:10.3390/life8030025)
Supplement: Supplementary file 1 [file life-08-00025-s001.zip › life-305093.R1_RevisedManuscript_SupplementaryFiles/SupplementaryFile2_Script02.pdf]

```bash
#!/bin/bash

# AUTHOR="Michael Gruenstaeudl, PhD"
# COPYRIGHT="Copyright (C) 2016-2018 $AUTHOR"
# CONTACT="m.gruenstaeudl@fu-berlin.de"
# VERSION="2018.04.03.1800"
# USAGE="bash Script2.sh $INF1 $INF2 $LOG"

########################################################################
# SUPPLEMENTARY FILE 2                                                 #
# Bash script to conduct quality filtering of Illumina reads and to    #
# quantify the loss of reads through quality filtering.                #
########################################################################

# Check if sufficient commandline parameters
numArgmts=$#
if [ ! $numArgmts -eq 3 ]; then
    echo "ERROR | Incorrect number of commandline parameters" >&2
    exit 1
fi

# Check if input files exist
for v in "$@"; do
if [ ! -f "$v" ]; then
    echo "ERROR | File not found: $v" >&2
    exit 1
fi
done

# Check if dependencies exist
DEPS=(fastq_quality_filter) # fastq_quality_filter of the 'FASTX Toolkit'
for d in "${DEPS[@]}"; do
if ! [ -x "$(command -v $d)" ]; then
  echo "Error: $d is not installed" >&2
  exit 1
fi
done

# Assigning commandline arguments
INF1=$1
INF2=$2
LOG=$3

# Defining temporary files and outfiles
OUF1=${INF1%.intersect*}.Q30filt.fastq
OUF2=${INF2%.intersect*}.Q30filt.fastq

# Conducting quality filtering
fastq_quality_filter -q 30 -i $INF1 -o $OUF1
fastq_quality_filter -q 30 -i $INF2 -o $OUF2

# Counting reads after generating ordered intersection
VAR1=$(cat $OUF1 | grep "^@M" | wc -l)
VAR2=$(cat $OUF2 | grep "^@M" | wc -l)
if (($VAR1 == $VAR2)); then echo "SUCCESS"; else echo "FAIL"; fi

# Logging results
echo -e "\n# NUMBER OF READS AFTER QUALITY FILTERING" >> $LOG
echo -ne "$OUF1: " >> $LOG
LC_ALL=C printf "%'d\n" $VAR1 >> $LOG
echo -ne "$OUF2: " >> $LOG
LC_ALL=C printf "%'d\n" $VAR2 >> $LOG

#EOF
```
