# Supplementary material for: Bioinformatic Workflows for Generating Complete Plastid Genome Sequences—An Example from Cabomba (Cabombaceae) in the Context of the Phylogenomic Analysis of the Water-Lily Clade
Source: Life (Basel). 2018 Jun 21;8(3):25. doi: 10.3390/life8030025 (PMC6160935; doi:10.3390/life8030025)
Supplement: Supplementary file 1 [file life-08-00025-s001.zip › life-305093.R1_RevisedManuscript_SupplementaryFiles/SupplementaryFile3_Script03.pdf]

```
#!/bin/bash

# AUTHOR="Michael Gruenstaeudl, PhD"
# COPYRIGHT="Copyright (C) 2016-2018 $AUTHOR"
# CONTACT="m.gruenstaeudl@fu-berlin.de"
# VERSION="2018.04.03.1800"
# USAGE="bash Script3.sh $INF1 $INF2 $LOG $IOGA $REF_DB $SAMPLE"

########################################################################
# SUPPLEMENTARY FILE 3                                                 #
# Bash script to automate the genome assembly process and to quantify  #
# assembly success.                                                    #
########################################################################

# Check if sufficient commandline parameters
numArgmts=$#
if [ ! $numArgmts -eq 6 ]; then
    echo "ERROR | Incorrect number of commandline parameters" >&2
    exit 1
fi

# Check if input files exist
# ${@:1:5} means all input arguments except third one
for v in "${@:1:5}"; do
if [ ! -f "$v" ]; then
    echo "ERROR | File not found: $v" >&2
    exit 1
fi
done

# Check if dependencies exist
DEPS=(python2.7)
for d in "${DEPS[@]}"; do
if ! [ -x "$(command -v $d)" ]; then
  echo "Error: $d is not installed" >&2
  exit 1
fi
done

# Assigning commandline arguments
INF1=$1
INF2=$2
LOG=$3
IOGA=$4
REF_DB=$5
SAMPLE=$6

# Conducting assembly
python2.7 $IOGA --reference $REF_DB --name $SAMPLE --forward $INF1 --reverse $INF2 --threads 4 --verbose >> ${SAMPLE}.assembly.log 2>&1
if [ -f ${SAMPLE}.final/${SAMPLE}.soap.ctg.fasta ]; then
    cp ${SAMPLE}.final/${SAMPLE}.soap.ctg.fasta ${SAMPLE}.soap.ctg.fasta
fi

# Counting final contigs ater assembly
VAR1=$(grep "^>" ${SAMPLE}.soap.ctg.fasta | wc -l)
VAR2=$(grep "Average coverage" ${SAMPLE}.assembly.log | tail -n1 | awk '{print $3}')

# Logging results
echo -e "\n# ASSEMBLY STATISTICS" >> $LOG
echo -ne "Number of contigs: " >> $LOG
LC_ALL=C printf "%'d\n" $VAR1 >> $LOG
echo -ne "Average coverage: " >> $LOG
#LC_ALL=C printf "%'.2f\n" $VAR2 >> $LOG
#LANG="de_DE" printf "%'.2f\n" $VAR2 >> $LOG
echo $VAR2 >> $LOG

#EOF
```
