# Supplementary material for: Bioinformatic Workflows for Generating Complete Plastid Genome Sequences—An Example from Cabomba (Cabombaceae) in the Context of the Phylogenomic Analysis of the Water-Lily Clade
Source: Life (Basel). 2018 Jun 21;8(3):25. doi: 10.3390/life8030025 (PMC6160935; doi:10.3390/life8030025)
Supplement: Supplementary file 1 [file life-08-00025-s001.zip › life-305093.R1_RevisedManuscript_SupplementaryFiles/SupplementaryFile4_Script04.pdf]

```bash
#!/bin/bash

# AUTHOR="Michael Gruenstaeudl, PhD"
# COPYRIGHT="Copyright (C) 2016-2018 $AUTHOR"
# CONTACT="m.gruenstaeudl@fu-berlin.de"
# VERSION="2018.04.03.1800"
# USAGE="bash Script4.sh $FINAL_ASMBLY $LOG"

########################################################################
# SUPPLEMENTARY FILE 4                                                 #
# Bash script to automate the confirmation of IR boundaries via        #
# self-blasting.                                                       #
########################################################################

# Check if sufficient commandline parameters
numArgmts=$#
if [ ! $numArgmts -eq 2 ]; then
    echo "ERROR | Incorrect number of commandline parameters" >&2
    exit 1
fi

# Check if input files exist
for v in "$@"; do
if [ ! -f "$v" ]; then
    echo "ERROR | File not found: $v" >&2
    exit 1
fi
done

# Check if dependencies exist
DEPS=(blastn)
for d in "${DEPS[@]}"; do
if ! [ -x "$(command -v $d)" ]; then
  echo "Error: $d is not installed" >&2
  exit 1
fi
done

# Assigning commandline arguments
FINAL_ASMBLY=$1
LOG=$2

# Logging results
echo -en "\n# SELF-BLASTING" >> $LOG
echo -e "\n# alignment length, IRa start, IRa end, IRb end, IRb start" >> $LOG

# Self-blasting
blastn -query $FINAL_ASMBLY -subject $FINAL_ASMBLY -outfmt 7 -strand 'both' | awk '{
 if ($4 > 10000 && $4 < 50000) print $4, $7, $8, $9, $10}' >> $LOG

#EOF
```
