# Supplementary material for: Bioinformatic Workflows for Generating Complete Plastid Genome Sequences—An Example from Cabomba (Cabombaceae) in the Context of the Phylogenomic Analysis of the Water-Lily Clade
Source: Life (Basel). 2018 Jun 21;8(3):25. doi: 10.3390/life8030025 (PMC6160935; doi:10.3390/life8030025)
Supplement: Supplementary file 1 [file life-08-00025-s001.zip › life-305093.R1_RevisedManuscript_SupplementaryFiles/SupplementaryFile5_Script05.pdf]

```bash
#!/bin/bash

# AUTHOR="Michael Gruenstaeudl, PhD"
# COPYRIGHT="Copyright (C) 2016-2018 $AUTHOR"
# CONTACT="m.gruenstaeudl@fu-berlin.de"
# VERSION="2018.04.03.1800"
# USAGE="bash Script5.sh $INF1 $INF2 $FINAL_ASMBLY $LOG $SAMPLE"

########################################################################
# SUPPLEMENTARY FILE 5                                                 #
# Bash script to automatically map the quality-filtered reads against  #
# the final assembly and to extract the mapped paired reads from the   #
# quality-filtered read files. This script also compiles a series of   #
# mapping statistics to describe the mapping process.                  #
########################################################################

# Check if sufficient commandline parameters
numArgmts=$#
if [ ! $numArgmts -eq 5 ]; then
    echo "ERROR | Incorrect number of commandline parameters" >&2
    exit 1
fi

# Check if input files exist
# ${@:1:4} means all input arguments except fifth one
for v in "${@:1:4}"; do
if [ ! -f "$v" ]; then
    echo "ERROR | File not found: $v" >&2
    exit 1
fi
done

# Check if dependencies exist
DEPS=(bowtie2-build bowtie2 samtools bedtools)
for d in "${DEPS[@]}"; do
if ! [ -x "$(command -v $d)" ]; then
  echo "Error: $d is not installed" >&2
  exit 1
fi
done

# Assigning commandline arguments
INF1=$1
INF2=$2
FINAL_ASMBLY=$3
LOG=$4
SAMPLE=$5

################################################################################

## Mapping of all quality-filtered reads to the final assemly to infer number
## of reads that map to assembled genome

# Defining temporary files and outfiles
REF_BASE=$(basename $FINAL_ASMBLY)
REF_FN=${REF_BASE%.fasta*}
OUT_STEM=${SAMPLE}.MappedAgainst.${REF_FN}
OUT1_FLE=${OUT_STEM}.sam
OUT1_STAT1=${OUT_STEM}.part1.stats
OUT1_STAT2=${OUT_STEM}.part2.stats

# Build reference database
mkdir -p db
bowtie2-build $REF_BASE db/$REF_FN > ${OUT_STEM}.refdb.log

# Mapping reads with bowtie2
bowtie2 -x db/$REF_FN -1 $INF1 -2 $INF2 -S $OUT1_FLE 2>> $OUT1_STAT1

# Extracting mapping statistics
samtools flagstat $OUT1_FLE >> $OUT1_STAT2

################################################################################
```

```
## Extracting successfully mapped pairs

# Defining temporary files and outfiles
TMP1=${OUT_STEM}.extracted.sam
TMP2=${OUT_STEM}.sorted.sam
OUT2_FLE=${OUT_STEM}.fastq

# Extracting only paired mapped reads
#samtools view -b -f 0x10 -f 0x20 -F 0x40 $INF > $TMP1
samtools view -b -F12 $OUT1_FLE > $TMP1  # See: https://www.biostars.org/p/56246/
# Samtool flags:
# 1  The read is one of a pair
# 2  The alignment is one end of a proper paired-end alignment

# Sorting BAM file by read header (necessary for the command `bedtools bamtofastq`)
samtools sort -n $TMP1 > $TMP2

# Extracting fastq sequences
bedtools bamtofastq -i $TMP2 -fq $OUT2_FLE

# Separating successfully mapped paired reads into an R1 file and an R2 file
cat $OUT2_FLE | awk -v n=4 'BEGIN{f=2} { if((NR-1)%n==0){f=1-f}; print > "out_R" f "
.fastq"}'
mv out_R-1.fastq ${OUT_STEM}_R1.fastq
mv out_R2.fastq ${OUT_STEM}_R2.fastq

# File hygiene
rm $TMP1
rm $TMP2

#################################################################################

# Logging results
echo -e "\n# NUMBER OF READS/PAIRS THAT MAPPED TO REFERENCE GENOME" >> $LOG
echo -e "Name of reference genome: $REF_FN" >> $LOG

echo -ne "\nN of reads that paired: " >> $LOG
R_VAR1=$(head -n1 $OUT1_STAT2 | awk '{print $1}')
LC_ALL=C printf "%'d\n" $R_VAR1 >> $LOG
echo -ne "N of reads that mapped (incl. cases where only R1 or R2 mapped): " >> $LOG
R_VAR2=$(grep " mapped (" $OUT1_STAT2 | awk '{print $1}')
LC_ALL=C printf "%'d\n" $R_VAR2 >> $LOG
echo -ne "P of reads that mapped (incl. cases where only R1 or R2 mapped): " >> $LOG
R_VAR3=$(grep " mapped (" $OUT1_STAT2 | awk -F'(' '{print $2}' | awk '{print $1}')
echo "$R_VAR3" >> $LOG
echo -ne "N of reads that mapped (excl. cases where only R1 or R2 mapped): " >> $LOG
R_VAR4=$(grep " properly paired (" $OUT1_STAT2 | awk '{print $1}')
LC_ALL=C printf "%'d\n" $R_VAR4 >> $LOG
echo -ne "P of reads that mapped (excl. cases where only R1 or R2 mapped): " >> $LOG
R_VAR5=$(grep " properly paired (" $OUT1_STAT2 | awk -F'(' '{print $2}' | awk '{prin
t $1}')
echo "$R_VAR5" >> $LOG

echo -ne "\nN of pairs: " >> $LOG
P_VAR1=$(grep "were paired" $OUT1_STAT1 | awk '{print $1}')
LC_ALL=C printf "%'d\n" $P_VAR1 >> $LOG
echo -ne "N of pairs that mapped exactly one time: " >> $LOG
P_VAR2=$(grep "aligned concordantly exactly 1 time" $OUT1_STAT1 | awk '{print $1}')
LC_ALL=C printf "%'d\n" $P_VAR2 >> $LOG
echo -ne "P of pairs that mapped exactly one time: " >> $LOG
LC_ALL=C printf '%.2f%%\n' "$(echo "scale=4; ($P_VAR2/$P_VAR1)*100" | bc)" >> $LOG
echo -ne "N of pairs that mapped one or more times: " >> $LOG
P_VAR3=$(grep -A1 "aligned concordantly exactly 1 time" $OUT1_STAT1 | awk '{print $1
}' | paste -sd+ | bc)
LC_ALL=C printf "%'d\n" $P_VAR3 >> $LOG
echo -ne "P of pairs that mapped one or more times: " >> $LOG
LC_ALL=C printf '%.2f%%\n' "$(echo "scale=4; ($P_VAR3/$P_VAR1)*100" | bc)" >> $LOG

#################################################################################

#EOF
```
