# Supplementary material for: Bioinformatic Workflows for Generating Complete Plastid Genome Sequences—An Example from Cabomba (Cabombaceae) in the Context of the Phylogenomic Analysis of the Water-Lily Clade
Source: Life (Basel). 2018 Jun 21;8(3):25. doi: 10.3390/life8030025 (PMC6160935; doi:10.3390/life8030025)
Supplement: Supplementary file 1 [file life-08-00025-s001.zip › life-305093.R1_RevisedManuscript_SupplementaryFiles/SupplementaryFile6_Script06.pdf]

```bash
#!/bin/bash

# AUTHOR="Michael Gruenstaeudl, PhD"
# COPYRIGHT="Copyright (C) 2016-2018 $AUTHOR"
# CONTACT="m.gruenstaeudl@fu-berlin.de"
# VERSION="2018.04.03.1800"
# USAGE="bash Script6.sh $INF_MAPPED_R1 $INF_MAPPED_R2 $INF_SAM $LOG"

#########################################################################
# SUPPLEMENTARY FILE 6                                                  #
# Bash script to automatically generate assembly quality statistics    #
# (i.e., the mean read length of mapped reads and the number of         #
# nucleotides with coverage depth equal or greater than 20, 50 and     #
# 100).                                                                 #
#########################################################################

# Check if sufficient commandline parameters
numArgmts=$#
if [ ! $numArgmts -eq 4 ]; then
    echo "ERROR | Incorrect number of commandline parameters" >&2
    exit 1
fi

# Check if input files exist
for v in "$@"; do
if [ ! -f "$v" ]; then
    echo "ERROR | File not found: $v" >&2
    exit 1
fi
done

# Check if dependencies exist
DEPS=(bowtie2 samtools bedtools)
for d in "${DEPS[@]}"; do
if ! [ -x "$(command -v $d)" ]; then
  echo "Error: $d is not installed" >&2
  exit 1
fi
done

# Assigning commandline arguments
INF_MAPPED_R1=$1
INF_MAPPED_R2=$2
INF_SAM=$3
LOG=$4

#################################################################################

## Calculating mean read length

# Defining outfiles
OUF1=${INF_MAPPED_R1%.fastq*}_readLen.txt
OUF2=${INF_MAPPED_R2%.fastq*}_readLen.txt

# Calculate read lengths of R1 and R2
grep --no-group-separator -A1 "^@M" $INF_MAPPED_R1 | grep -v "^@M" | awk '{print length($0)}' > $OUF1
grep --no-group-separator -A1 "^@M" $INF_MAPPED_R2 | grep -v "^@M" | awk '{print length($0)}' > $OUF2

# Logging results
echo -e "\n# MEAN READ LENGTH (R1 PLUS R2)" >> $LOG
paste -d '\t' $OUF1 $OUF2 | awk '{ total += $1+$2 } END { print total/NR }' >> $LOG

#################################################################################

## Calculating N of bases with coverage depth equal or greater than to x, where x={20,50,100}

# Defining temporary files and outfiles
INF_BAM=${INF_SAM%.sam*}.bam
TMP_SRTD=${INF_BAM%.bam*}.sorted.bam
TMP_GNM=${INF_BAM%.bam*}.genome
```

```
OUF3=${INF_BAM%.bam*}.genomecov

# Generating genome dummy file
touch $TMP_GNM
REF_LEN=$(head $INF_SAM | grep "^@SQ" | awk -F'LN:' '{print $2}')
echo -e "plastid\t$REF_LEN" >> $TMP_GNM

# Convert SAM to BAM
samtools view -bS $INF_SAM > $INF_BAM

# Sorting BAM file by position
samtools sort $INF_BAM > $TMP_SRTD

# Report per-base genome coverage
bedtools genomecov -ibam $TMP_SRTD -g $TMP_GNM -d > $OUF3

# Logging results
echo -e "\n# COVERAGE DEPTH STATISTICS" >> $LOG

echo -ne "Genome size (bp): " >> $LOG
GNM_SZE=$(awk '{print $2}' $TMP_GNM)
LC_ALL=C printf "%'d\n" $GNM_SZE >> $LOG

echo -ne "N of bases with coverage depth greater than 20-fold: " >> $LOG
COV_DEP_20=$(awk '$3>20' $OUF3 | wc -l)
LC_ALL=C printf "%'d\n" $COV_DEP_20 >> $LOG
echo -ne "P of bases with coverage depth greater than 20-fold: " >> $LOG
LC_ALL=C printf '%.2f\n' "$(echo "scale=4; ($COV_DEP_20/$GNM_SZE)*100" | bc)" >> $LO
G

echo -ne "N of bases with coverage depth greater than 50-fold: " >> $LOG
COV_DEP_50=$(awk '$3>50' $OUF3 | wc -l)
LC_ALL=C printf "%'d\n" $COV_DEP_50 >> $LOG
echo -ne "P of bases with coverage depth greater than 50-fold: " >> $LOG
LC_ALL=C printf '%.2f\n' "$(echo "scale=4; ($COV_DEP_50/$GNM_SZE)*100" | bc)" >> $LO
G

echo -ne "N of bases with coverage depth greater than 100-fold: " >> $LOG
COV_DEP_100=$(awk '$3>100' $OUF3 | wc -l)
LC_ALL=C printf "%'d\n" $COV_DEP_100 >> $LOG
echo -ne "P of bases with coverage depth greater than 100-fold: " >> $LOG
LC_ALL=C printf '%.2f\n' "$(echo "scale=4; ($COV_DEP_100/$GNM_SZE)*100" | bc)" >> $L
OG

# File hygiene
rm $TMP_SRTD
rm $TMP_GNM

#EOF
```
