# Supplementary material for: Bioinformatic Workflows for Generating Complete Plastid Genome Sequences—An Example from Cabomba (Cabombaceae) in the Context of the Phylogenomic Analysis of the Water-Lily Clade
Source: Life (Basel). 2018 Jun 21;8(3):25. doi: 10.3390/life8030025 (PMC6160935; doi:10.3390/life8030025)
Supplement: Supplementary file 1 [file life-08-00025-s001.zip › life-305093.R1_RevisedManuscript_SupplementaryFiles/SupplementaryFile7_Script07.pdf]

```
#!/bin/bash

# AUTHOR="Michael Gruenstaeudl, PhD"
# COPYRIGHT="Copyright (C) 2016-2018 $AUTHOR"
# CONTACT="m.gruenstaeudl@fu-berlin.de"
# VERSION="2018.04.05.1800"
# USAGE="bash Script7.sh $INF $FINAL_ASMBLY"

########################################################################
# SUPPLEMENTARY FILE 7                                                 #
# Bash script to convert the plain text summary of the annotations     #
# generated by DOGMA to a GFF file.                                    #
########################################################################

# Check if sufficient commandline parameters
numArgmts=$#
if [ ! $numArgmts -eq 2 ]; then
    echo "ERROR | Incorrect number of commandline parameters" >&2
    exit 1
fi

# Check if input files exist
for v in "$@"; do
if [ ! -f "$v" ]; then
    echo "ERROR | File not found: $v" >&2
    exit 1
fi
done

# Check if dependencies exist
DEPS=(python2.7 perl)
for d in "${DEPS[@]}"; do
if ! [ -x "$(command -v $d)" ]; then
  echo "Error: $d is not installed" >&2
  exit 1
fi
done

# Assigning commandline arguments
INF=$1
FINAL_ASMBLY=$2

################################################################################

# Defining temporary files and outfiles
OUT_STEM=${INF%.txt*}
OUT_FLE=${OUT_STEM}.gff
TMP1=${OUT_STEM}_part1.tmp
TMP2=${OUT_STEM}_part2.tmp

# Main operations
SEQNAME=$(head -n1 $FINAL_ASMBLY | tr -d ">")
NLINES=$(($(wc -l $INF | awk '{print $1}')-1))
printf "$SEQNAME\tDOGMA\tmotif\n%.0s" $(seq 1 $NLINES) > $TMP1
tail -n +2 $INF | awk '{print $1, $2, ".", $4, ".", "Name="$3}' > $TMP2
paste ${OUT_STEM}_part*.tmp | column -t -s $'\t' > $OUT_FLE
sed -i 's/ \+ /\t/g' $OUT_FLE
sed -i 's/ /\t/g' $OUT_FLE
echo "##FASTA" >> $OUT_FLE
cat $FINAL_ASMBLY >> $OUT_FLE


# File hygiene
rm $TMP1
rm $TMP2

#EOF
```
