# Supplementary material for: Bioinformatic Workflows for Generating Complete Plastid Genome Sequences—An Example from Cabomba (Cabombaceae) in the Context of the Phylogenomic Analysis of the Water-Lily Clade
Source: Life (Basel). 2018 Jun 21;8(3):25. doi: 10.3390/life8030025 (PMC6160935; doi:10.3390/life8030025)
Supplement: Supplementary file 1 [file life-08-00025-s001.zip › life-305093.R1_RevisedManuscript_SupplementaryFiles/SupplementaryFile8_Script08.pdf]

```
#!/bin/bash

# AUTHOR="Michael Gruenstaeudl, PhD"
# COPYRIGHT="Copyright (C) 2016-2018 $AUTHOR"
# CONTACT="m.gruenstaeudl@fu-berlin.de"
# VERSION="2018.04.05.1800"
# USAGE="bash Script8.sh $INF_CPGAVAS $INF_DOGMA $FINAL_ASMBLY $IRB $IRA"

#########################################################################
# SUPPLEMENTARY FILE 8                                                  #
# Bash script to combine the annotations produced by the annotation    #
# servers DOGMA (http://dogma.ccbb.utexas.edu/) and cpGAVAS             #
# (http://www.herbalgenomics.org/cpgavas/)and to generate a union set  #
# of the combined annotations.                                          #
#########################################################################

# Check if sufficient commandline parameters
numArgmts=$#
if [ ! $numArgmts -eq 1 ]; then
    echo "ERROR | Incorrect number of commandline parameters" >&2
    exit 1
fi

# Check if input files exist
for v in "$@"; do
if [ ! -f "$v" ]; then
    echo "ERROR | File not found: $v" >&2
    exit 1
fi
done

# Check if dependencies exist
DEPS=( )
for d in "${DEPS[@]}"; do
if ! [ -x "$(command -v $d)" ]; then
  echo "Error: $d is not installed" >&2
  exit 1
fi
done

# Assigning commandline arguments
INF_CPGAVAS=$1
INF_DOGMA=$2
FINAL_ASMBLY=$3
IRB=$4
IRA=$5

#################################################################################

# Defining temporary files and outfiles
TMP1_DOGMA=${INF_DOGMA%.gff*}.SeqnameReplaced.gff
TMP2_DOGMA=${INF_DOGMA%.gff*}.split.
TMP2_CPGAVAS=${INF_CPGAVAS%.gff*}.split.
TMP3=${INF_CPGAVAS%.gff*}.combined.gff
OUTF=${INF_CPGAVAS%.gff*}.unionSet.gff

################################################
## Combining annotations of DOGMA and of cpGAVAS
################################################
SEQNAME_CPGAVAS=$(grep "^>" $INF_CPGAVAS | tr -d ">")
SEQNAME_DOGMA=$(grep "^>" $INF_DOGMA | tr -d ">")
# Replace sequence name in DOGMA.gff-file with sequence name of cpGAVAS.gff-file
sed "s/$SEQNAME_DOGMA/$SEQNAME_CPGAVAS/g" $INF_DOGMA > $TMP1_DOGMA
# Split cpGAVAS.gff-file at keyword "##FASTA"
csplit $INF_CPGAVAS --prefix=$TMP2_CPGAVAS -q '/^##FASTA/'
# Split DOGMA.gff-file at keyword "##FASTA"
csplit $TMP1_DOGMA --prefix=$TMP2_DOGMA -q '/^##FASTA/'
# Reassemble cpGAVAS.gff-file-part1, DOGMA.gff-file-part1 and cpGAVAS.gff-file-part2
cat "${TMP2_CPGAVAS}00" > $TMP3
cat "${TMP2_DOGMA}00" >> $TMP3
cat "${TMP2_CPGAVAS}01" >> $TMP3
```

```
##################
## Main operations
##################
grep -v "#" $TMP3 | sed 's/\t/_/4' > tmp
RANGES=$(sort -k4 -n tmp | awk '{print $4}' | uniq)
mkdir -p temp
for i in $RANGES; do
  grep $'\t'$i$'\t' tmp > temp/l$i.txt;
done
for f in $(ls temp/l*.txt); do
  grep -i -m 1 $'\t'CDS$'\t' $f > tmp
  grep -i -m 1 $'\t'cds$'\t' $f > tmp
  grep -i -m 1 $'\t'exon$'\t' $f >> tmp
  grep -i -m 1 $'\t'gene$'\t' $f >> tmp
  grep -i -m 1 $'\t'intron$'\t' $f >> tmp
  grep -i -m 1 $'\t'tRNA_primary_transcript$'\t' $f >> tmp
  grep -i -m 1 $'\t'tRNA$'\t' $f >> tmp
  grep -i -m 1 $'\t'rRNA$'\t' $f >> tmp
  grep -i -m 1 $'\t'motif$'\t' $f >> tmp
  grep -i $'\t'repeat_region$'\t' $f >> tmp
  grep -i $'\t'misc_feature$'\t' $f >> tmp
  grep -i $'\t'region$'\t' $f >> tmp
  mv tmp $f
done
cat temp/l*.txt > tmp
awk -v FPAT='("[^"]*"|[^[:blank:]"]+)+' -v OFS='\t' '{gsub("_", "\t", $4); print $0}
' tmp > tmp2 && mv tmp2 tmp
sort -k4,5 -n tmp > tmp2 && mv tmp2 tmp
awk -v FPAT='("[^"]*"|[^[:blank:]"]+)+' -v OFS='\t' '{$2="maker"; print}' tmp > tmp2
 && mv tmp2 tmp
awk -v FPAT='("[^"]*"|[^[:blank:]"]+)+' -v OFS='\t' '{$6="."; print}' tmp > tmp2 &&
mv tmp2 tmp
sed -i 's/\;ID\=.*//' tmp
awk -v FPAT='("[^"]*"|[^[:blank:]"]+)+' -v OFS='\t' '{$9=$9";Annotation="$9; print}'
 tmp > tmp2 && mv tmp2 tmp
sed -i 's/\=Name\=/\=/' tmp
sed -i 's/\(Name=trn.\)/\1-/' tmp
sed -i 's/\(Annotation=trn.\)/\1-/' tmp
sed -i 's/--/-/' tmp

python2.7 - <<EOF
import re
with open("tmp", "r+") as f:
  lines = f.read().splitlines()
  outL = []
  kw = "=trn"
  for l in lines:
    if kw in l:
      pos = [m.end()+2 for m in re.finditer("=trn", l)]
      for p in pos:
        l = l[:p] + l[p:p+3].replace("U","T") + l[p+3:]
      outL.append(l)
    else:
      outL.append(l)
  f.seek(0)
  f.write('\n'.join(outL))
  f.truncate()
EOF

sed -i 's/trnf\-MCAU/trnf\-MCAT/g' tmp

python2.7 - <<EOF
with open("tmp", "r+") as f:
  lines = f.read().splitlines()
  outL = []
  for l in lines:
    annotyp = l.split("\t")[2]
    if l[-5:] == " gene" and annotyp == "gene":
      outL.append(l[:-5])
    elif l[-7:] == " intron" and annotyp == "intron":
      outL.append(l[:-7])
    elif l[-7:-1] == " exon " and annotyp == "exon" and "number=" not in l:
      outL.append(l[:-7]+";number="+l[-1:])
```

```
    else:
      outL.append(l)
  f.seek(0)
  f.write('\n'.join(outL))
  f.truncate()
EOF

grep -v "#" $FINAL_ASMBLY > tmp3

python2.7 - <<EOF
import re
with open("tmp3", "r") as f:
  lines = f.read().splitlines()
  annoDict = {}
  for l in lines:
    li = re.split(r'(;|\t)', l)
    #li = re.split(r'[;\s]\s*', l)
    if any("Name=" and "product=" in e for e in li):
      n = [e for e in li if "Name=" in e][0]
      a_name = n[n.find("Name=")+len("Name="):]
      p = [e for e in li if "product=" in e][0]
      a_prod = p[p.find("product=")+len("product="):]
      annoDict[a_name] = a_prod
  f.close()
with open("tmp", "r+") as f:
  lines = f.read().splitlines()
  outL = []
  for l in lines:
    annotyp = l.split()[2]
    if annotyp != "intron":
      li = re.split(r'(;|\t)', l)
      #li = re.split(r'[;\s]\s*', l)
      if any("Name=" in e for e in li):
        n = [e for e in li if "Name=" in e][0]
        a_name = n[n.find("Name=")+len("Name="):]
        kw = n[n.find("Name="):]
        if a_name in annoDict.keys() and kw in l:
          # "product" in tfl must start w. small letter
          nl = l + ";product=" + annoDict[a_name]
          outL.append(nl)
        else:
          outL.append(l)
    else:
      outL.append(l)
  f.seek(0)
  f.write('\n'.join(outL))
  f.truncate()
EOF

awk -v FPAT='("[^"]*"|[^[:blank:]"]+)+' -v OFS='\t' '{if ($3=="intron") $7="."; prin
t}' tmp > tmp2 && mv tmp2 tmp
sed -i 's/tRNA_primary_transcript/tRNA/' tmp

python2.7 - <<EOF
ira = $IRA
ira = [int(x) for x in list(ira)]
irb = $IRB
irb = [int(x) for x in list(irb)]
with open("tmp", "r+") as f:
  lines = f.read().splitlines()
  outL = []
  for l in lines:
    li = l.split("\t")
    tags = "".join(li[8:]).split(";")
    tagD = {}
    for t in tags:
      if "=" in t:
        i = t.split("=")
        tagD[i[0]] = i[1] # Dict. saves only unique keys
    startp = int(li[3])
    stopp = int(li[4])
    if min(ira) <= startp <= max(ira) or min(ira) <= stopp <= max(ira):
      tagD["Name"] = tagD["Name"] + "_IRa"
```

```
    if min(irb) <= startp <= max(irb) or min(irb) <= stopp <= max(irb):
      tagD["Name"] = tagD["Name"] + "_IRb"
    #del tagD["createdby"]
    tagL = []
    for k,v in tagD.items():
      tagL.append(k+"="+v)
    nl = "\t".join(li[:8]) + "\t" + ";".join(tagL)
    outL.append(nl)
  f.seek(0)
  f.write('\n'.join(outL))
  f.truncate()
EOF

python2.7 - <<EOF
with open("tmp", "r+") as f:
  lines = f.read().splitlines()
  outL = []
  for l in lines:
    li = l.split("\t")
    tags = "".join(li[8:]).split(";")
    tagD = {}
    for t in tags:
      if "=" in t:
        i = t.split("=")
        tagD[i[0]] = i[1] # Dict. saves only unique keys
    if li[2] == "exon" and "number" in tagD:
      del tagD["number"]
    tagL = []
    for k,v in tagD.items():
      tagL.append(k+"="+v)
    nl = "\t".join(li[:8]) + "\t" + ";".join(tagL)
    outL.append(nl)
  f.seek(0)
  f.write('\n'.join(outL))
  f.truncate()
EOF

sed -i 's/--/-/' tmp
sed -i 's/exon1/_exon_1/g' tmp
sed -i 's/exon1/_exon_2/g' tmp
sed -i 's/NCBIFeatureKey/NCBI Feature Key/g' tmp
sed -i 's/NCBIJoinType/NCBI Join Type/g' tmp
awk '{print $0";"}' tmp > tmp2 && mv tmp2 tmp

perl -pi -le 's/(Annotation=.*?)CDS(.*?;)/$1$2/' tmp
perl -pi -le 's/(Annotation=.*?)gene(.*?;)/$1$2/' tmp
perl -pi -le 's/(Annotation=.*?)exon1(.*?;)/$1$2/' tmp
perl -pi -le 's/(Annotation=.*?)exon2(.*?;)/$1$2/' tmp
perl -pi -le 's/(Annotation=.*?)exon3(.*?;)/$1$2/' tmp
perl -pi -le 's/(Annotation=.*?)exon(.*?;)/$1$2/' tmp
perl -pi -le 's/(Annotation=.*?)intron1(.*?;)/$1$2/' tmp
perl -pi -le 's/(Annotation=.*?)intron2(.*?;)/$1$2/' tmp
perl -pi -le 's/(Annotation=.*?)intron3(.*?;)/$1$2/' tmp
perl -pi -le 's/(Annotation=.*?)intron(.*?;)/$1$2/' tmp
perl -pi -le 's/(Annotation=.*?)tRNA(.*?;)/$1$2/' tmp

sed -i 's/ ;/;/g' tmp
awk '{print substr($0, 1, length($0)-1)}' tmp > tmp2 && mv tmp2 tmp

# Assemblying output file
grep "^#" $TMP3 | grep -v "FASTA" > $OUTF
cat tmp >> $OUTF
grep single_copy_region $TMP3 >> $OUTF
cat ${TMP2_CPGAVAS}01 >> $OUTF


# File hygiene
rm tmp
#rm tmp2
rm tmp3
rm $TMP1_DOGMA
rm ${TMP2_DOGMA}0*
rm ${TMP2_CPGAVAS}0*
```

```
rm $TMP3
rm -r temp/

#EOF
```
