# Supplementary material for: Bioinformatic Workflows for Generating Complete Plastid Genome Sequences—An Example from Cabomba (Cabombaceae) in the Context of the Phylogenomic Analysis of the Water-Lily Clade
Source: Life (Basel). 2018 Jun 21;8(3):25. doi: 10.3390/life8030025 (PMC6160935; doi:10.3390/life8030025)
Supplement: Supplementary file 1 [file life-08-00025-s001.zip › life-305093.R1_RevisedManuscript_SupplementaryFiles/SupplementaryFile9_Script09.pdf]

```python
#!/usr/bin/env python2.7
''' Python script to extract and align coding regions across a set of input genomes
in GenBank format '''

###############
# AUTHOR INFO #
###############
__author__ = 'Michael Gruenstaeudl, PhD'
__contact__ = "m.gruenstaeudl@fu-berlin.de"
__copyright__ = 'Copyright (C) 2016-2018 ' + __author__
__info__ = 'Extract and align coding regions across a set of input genomes'
__version__ = '2018.04.30.1630'

#####################
# IMPORT OPERATIONS #
#####################
from Bio import SeqIO
from Bio.SeqRecord import SeqRecord
from Bio.Seq import Seq
from Bio import Alphabet
from Bio.Alphabet import IUPAC
from Bio import AlignIO  # For function 'AlignIO.convert'
from Bio.Nexus import Nexus  # For functions 'Nexus.combine' and 'Nexus.Nexus'
from Bio.Align.Applications import MafftCommandline
from StringIO import StringIO  # For file handles
import collections  # For 'collections.OrderedDict()'
import os
import subprocess
import sys

#############
# FUNCTIONS #
#############
def extract_collect_CDS(masterdict_nucl, masterdict_prot, inFn, fmt='genbank'):
    rec = SeqIO.read(inFn, fmt)
    for feature in rec.features:
        if feature.type == 'CDS':
            if 'gene' in feature.qualifiers:
                gene_name = feature.qualifiers['gene'][0]
                seq_name = gene_name + '_' + rec.name

                # Nucleotide sequences
                seq_obj = feature.extract(rec).seq
                seq_rec = SeqRecord(seq_obj, id=seq_name, name='', description='')
                if gene_name in masterdict_nucl.keys():
                    tmp = masterdict_nucl[gene_name]
                    tmp.append(seq_rec)
                    masterdict_nucl[gene_name] = tmp
                else:
                    masterdict_nucl[gene_name] = [seq_rec]

                # Protein sequences
                    # DON'T USE QUALIFIER 'TRANSLATION', AS GENES WITH INTRONS ARE C
OUNTED TWICE;
                    # KEEP TRANSLATING FROM EXTRACT
                    #if 'translation' in feature.qualifiers:
                    #    transl = feature.qualifiers['translation'][0]
                    #    seq_obj = Seq(transl, IUPAC.protein)
                    #else:
                    #    seq_obj = feature.extract(rec).seq.translate(table=11, cds=
True)
                seq_obj = feature.extract(rec).seq.translate(table=11)#, cds=True)
                seq_rec = SeqRecord(seq_obj, id=seq_name, name='', description='')
                if gene_name in masterdict_prot.keys():
                    tmp = masterdict_prot[gene_name]
                    tmp.append(seq_rec)
                    masterdict_prot[gene_name] = tmp
                else:
                    masterdict_prot[gene_name] = [seq_rec]

def remove_duplicates(my_dict):
    for k,v in my_dict.iteritems():
        idtags = []
```

```
        for counter, seqrec in enumerate(v):
            if seqrec.id in idtags:
                #v.pop(counter)
                del v[counter]
            else:
                idtags.append(seqrec.id)
        my_dict[k] = v
    #return my_dict

def main(inDir, outName, fext='.gb'):

    # MAKE OUTPUT FOLDER
    outDir = os.path.join(inDir, 'output_AlignmentFiles')
    if not os.path.exists(outDir):
        os.makedirs(outDir)

    # EXTRACT AND COLLECT CDS FROM RECORDS
    files = [f for f in os.listdir(inDir) if f.endswith(fext)]
    masterdict_nucl = collections.OrderedDict()
    masterdict_prot = collections.OrderedDict()
    for f in files:
        extract_collect_CDS(masterdict_nucl, masterdict_prot, os.path.join(inDir, f)
)

    # REMOVE ALL DUPLICATE ENTRIES (result of CDS with multiple exons)
    remove_duplicates(masterdict_nucl)
    remove_duplicates(masterdict_prot)
    # Note: Not sure why I have to run this removal twice, but not all
    #       duplicates are removed first time around.
    remove_duplicates(masterdict_nucl)
    remove_duplicates(masterdict_prot)

    # ALIGN AND WRITE TO FILE
    if masterdict_nucl.items():
        for k,v in masterdict_nucl.iteritems():
            outFn_unalign_nucl = os.path.join(outDir, 'nucl_'+k+'.unalign.fas')
            # Write unaligned nucleotide sequences
            with open(outFn_unalign_nucl, 'w') as hndl:
                SeqIO.write(v, hndl, 'fasta')
    if not masterdict_nucl.items():
        sys.exit('  ERROR: No items in nucleotide masterdictionary.')

    if masterdict_prot.items():
        for k,v in masterdict_prot.iteritems():
            outFn_unalign_prot = os.path.join(outDir, 'prot_'+k+'.unalign.fas')
            outFn_aligned_prot = os.path.join(outDir, 'prot_'+k+'.aligned.fas')
            # WRITE UNALIGNED PROTEIN SEQUENCES
            with open(outFn_unalign_prot, 'w') as hndl:
                SeqIO.write(v, hndl, 'fasta')
            # ALIGN SEQUENCES
            #import subprocess
            #subprocess.call(['mafft', '--auto', outFn_unalign_prot, '>', outFn_alig
ned_prot])
            mafft_cline = MafftCommandline(input=outFn_unalign_prot)
            stdout, stderr = mafft_cline()
            with open(outFn_aligned_prot, 'w') as hndl:
                hndl.write(stdout)
    if not masterdict_prot.items():
        sys.exit('  ERROR: No items in protein masterdictionary.')

    # BACK-TRANSLATION via Python script by Peter Cook
    # https://github.com/peterjc/pico_galaxy/tree/master/tools/align_back_trans
    for k,v in masterdict_prot.iteritems():
        outFn_unalign_nucl = os.path.join(outDir, 'nucl_'+k+'.unalign.fas')
        outFn_aligned_nucl = os.path.join(outDir, 'nucl_'+k+'.aligned.fas')
        outFn_aligned_prot = os.path.join(outDir, 'prot_'+k+'.aligned.fas')
        try:
            log = subprocess.check_output(['python2', 'align_back_trans.py', 'fasta'
, outFn_aligned_prot, outFn_unalign_nucl, outFn_aligned_nucl, '11'], stderr=subproce
ss.STDOUT)
        except:
            print '  ERROR: Error encountered during back-translation of', k
            #print log
```

```
    # IMPORT BACK-TRANSLATIONS AND CONCATENATE
    alignm_L = []
    for k in masterdict_prot.keys():
        aligned_nucl_fasta = os.path.join(outDir, 'nucl_'+k+'.aligned.fas')
        aligned_nucl_nexus = os.path.join(outDir, 'nucl_'+k+'.aligned.nex')
        # Convert from fasta to nexus
        try:
            alignm_fasta = AlignIO.read(aligned_nucl_fasta, 'fasta', alphabet=Alphab
et.generic_dna)
            hndl = StringIO()
            AlignIO.write(alignm_fasta, hndl, 'nexus')
            nexus_string = hndl.getvalue()
            nexus_string = nexus_string.replace('\n'+k+'_', '\ncombined_')  # IMPORT
ANT: Stripping the gene name from the sequence name
            alignm_nexus = Nexus.Nexus(nexus_string)
            alignm_L.append((k, alignm_nexus)) # Function 'Nexus.combine' needs a tu
ple.
        except:
            print '  ERROR: Cannot process alignment of', k

    # COMBINE THE NEXUS ALIGNMENTS (IN NO PARTICULAR ORDER)
    n_aligned_CDS = len(alignm_L)
    alignm_combined = Nexus.combine(alignm_L) # Function 'Nexus.combine' needs a tup
le.
    outFn_nucl_combined_nexus = os.path.join(inDir, outName+'_nucl_'+str(n_aligned_C
DS)+'combined.aligned.nex')
    alignm_combined.write_nexus_data(filename=open(outFn_nucl_combined_nexus, 'w'))
    #outFn_nucl_combined_fasta = os.path.join(outDir, outName+'_nucl_'+str(n_aligned
_CDS)+'combined.aligned.fas')
    #AlignIO.convert(outFn_nucl_combined_nexus, 'nexus', outFn_nucl_combined_fasta,
'fasta')

############
# ARGPARSE #
############
if __name__ == '__main__':
    import argparse
    parser = argparse.ArgumentParser(description="  --  ".join([__author__, __contac
t__, __copyright__, __info__, __version__]))

    # Required
    parser.add_argument('-i',
                        '--inp',
                        help='relative path to input directory; contains GenBank fil
es; Example: /path_to_input/',
                        default='/home/username/Desktop/',
                        required=True)

    parser.add_argument('-o',
                        '--oup',
                        help='sample name used for outfile; Example: `my_sample`',
                        default='my_sample',
                        required=True)

    # Optional
    parser.add_argument('-f',
                        '--fext',
                        help='File extension of input files',
                        default='.gb',
                        required=False)

    parser.add_argument('--version',
                        help='Print version information and exit',
                        action='version',
                        version='%(prog)s ' + __version__)

    args = parser.parse_args()

########
# MAIN #
########
    main(args.inp, args.oup, args.fext)
```
