# Supplementary figures and images for: Bioinformatic Workflows for Generating Complete Plastid Genome Sequences—An Example from Cabomba (Cabombaceae) in the Context of the Phylogenomic Analysis of the Water-Lily Clade
Source: Life (Basel). 2018 Jun 21;8(3):25. doi: 10.3390/life8030025 (PMC6160935; doi:10.3390/life8030025)

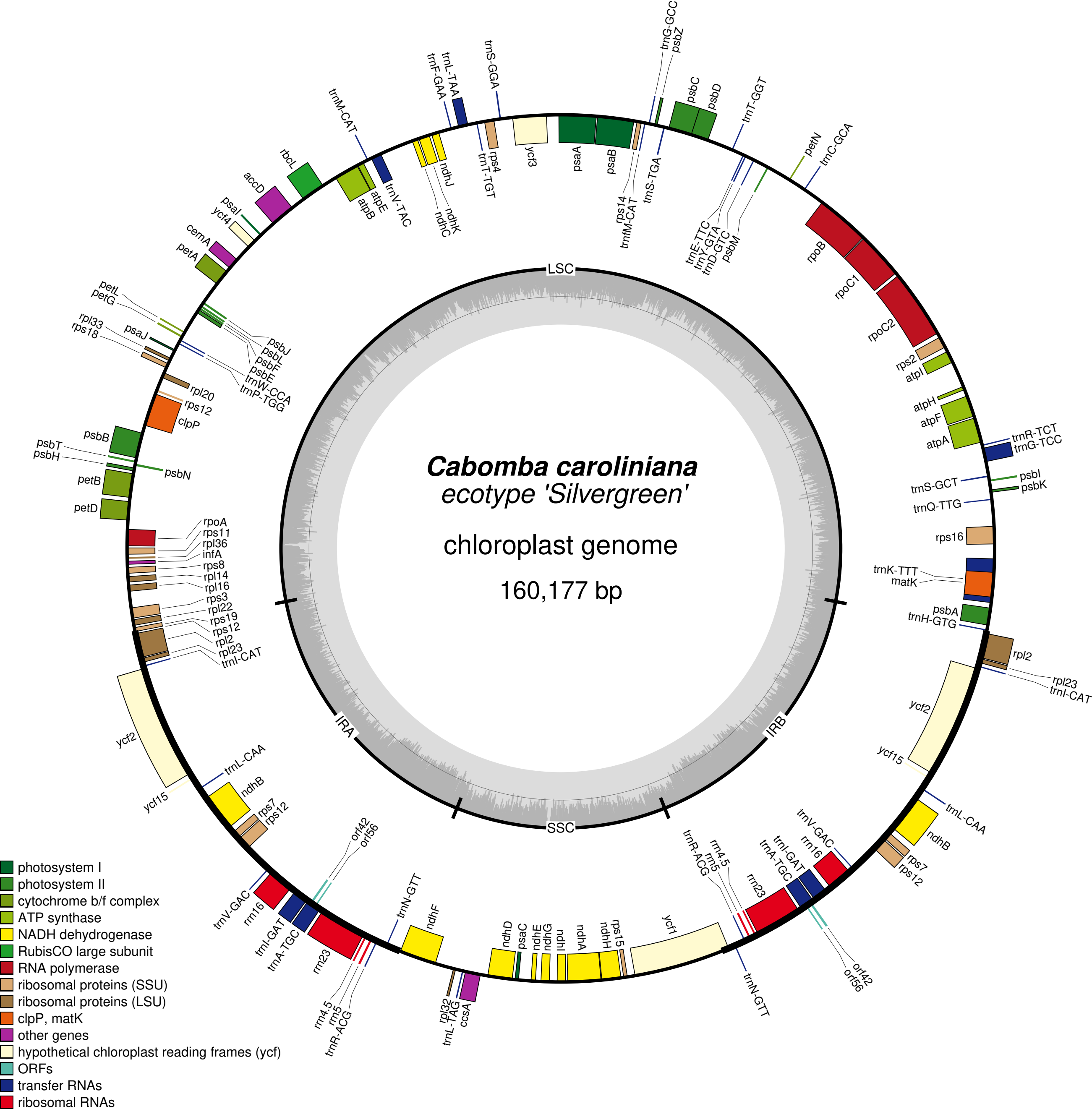

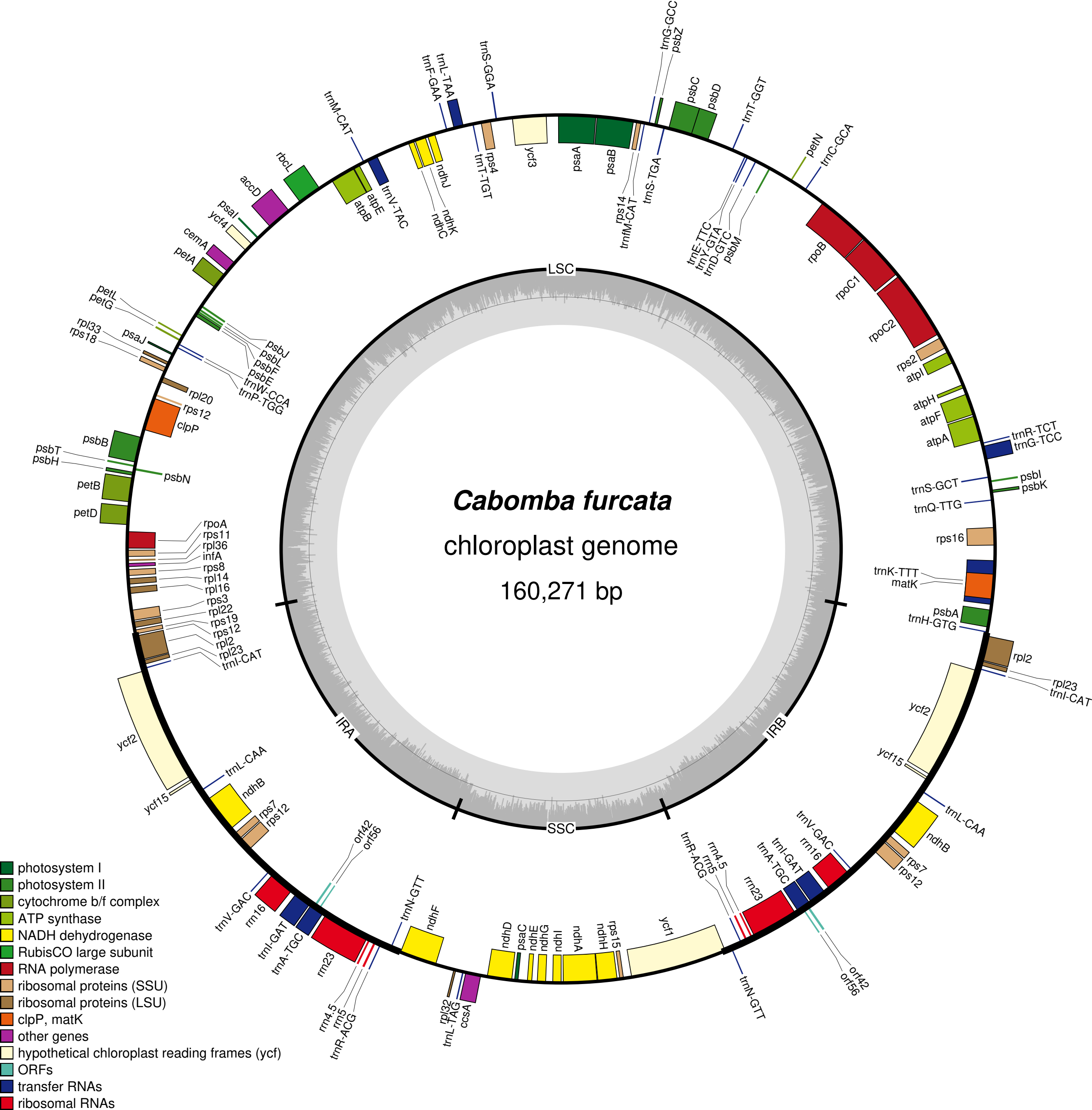

Supplement: Supplementary file 1 [file life-08-00025-s001.zip › life-305093.R1_RevisedManuscript_SupplementaryFiles/FigureS1.pdf]
